# An Osteoconductive Antibiotic Bone Eluting Putty with a Custom Polymer Matrix

**DOI:** 10.3390/polym8070247

**Published:** 2016-06-30

**Authors:** John Curley, Mohammad Raquibul Hasan, Jacob Larson, Benjamin D. Brooks, Qianhui Liu, Tanmay Jain, Abraham Joy, Amanda E. Brooks

**Affiliations:** 1 Department of Pharmaceutical Sciences, North Dakota State University, Fargo, ND 58105, USA; john.curley@ndsu.edu (J.C.); raquib.hasan@ndsu.edu (M.R.H.); 2 Department of Industrial and Manufacturing Engineering, North Dakota State University, Fargo, ND 58105, USA; jacob.s.larson@ndsu.edu; 3 Department of Electrical and Computer Engineering, North Dakota State University, Fargo, ND 58105, USA; ben@brooks.nu; 4 Department of Polymer Science, University of Akron, Akron, OH 44325, USA; ql17@zips.uakron.edu (Q.L.); tpj13@zips.uakron.edu (T.J.); abraham@uakron.edu (A.J.)

**Keywords:** osteomyelitis, controlled drug release, bone void filler, putty, pharmacokinetics, pendant-functionalized diol

## Abstract

With the rising tide of antibiotic resistant bacteria, extending the longevity of the current antibiotic arsenal is becoming a necessity. Developing local, controlled release antibiotic strategies, particularly for difficult to penetrate tissues such as bone, may prove to be a better alternative. Previous efforts to develop an osteoconductive local antibiotic release device for bone were created as solid molded composites; however, intimate contact with host bone was found to be critical to support host bone regrowth; thus, an osteocondconductive antibiotic releasing bone void filling putty was developed. Furthermore, a controlled releasing polymer matrix was refined using pendant-functionalized diols to provide tailorable pharmacokinetics. In vitro pharmacokinetic and bioactivity profiles were compared for a putty formulation with an analogous composition as its molded counterpart as well as four new pendant-functionalized polymers. A best-fit analysis of polymer composition in either small cylindrical disks or larger spheres revealed that the new pendant-functionalized polymers appear to release vancomycin via both diffusion and erosion regardless of the geometry of the putty. In silico simulations, a valuable technique for diffusion mediated controlled release models, will be used to confirm and optimize this property.

## 1. Introduction

Listed by the World Health Organization (WHO) as one of the top three threats to global public health [1], more than 2 million Americans suffer from an antibiotic-resistant infection [2], imposing an enormous economic burden (≈ $20 billion in direct healthcare costs [3]) on the world’s healthcare systems. Multidrug resistant bacteria are becoming almost universal, provoked by the pervasive and often extended use of antibiotics [4]; moreover, many of these once powerful antimicrobial drugs are now rendered impotent by inappropriate prescribing patterns [5,6,7] and poor patient compliance [4,8,9,10]. Almost 70% of nosocomial infections are linked to antibiotic resistant pathogens [11,12,13,14]. Beyond clinical practice, microbial characteristics as well as societal and technical changes have led to the frightening revelation that development of antibiotic resistance is almost virtually certain given enough time [15,16,17]. Thus, the rise in antibiotic-resistant bacterial strains is quickly outpacing drug discovery and several strategies beyond simply filling the new drug pipeline must be pursued. In the absence of such development, we are facing the return to a pre-antibiotic era, with drugs that are costly and only partially effective.

**Figure 1:**
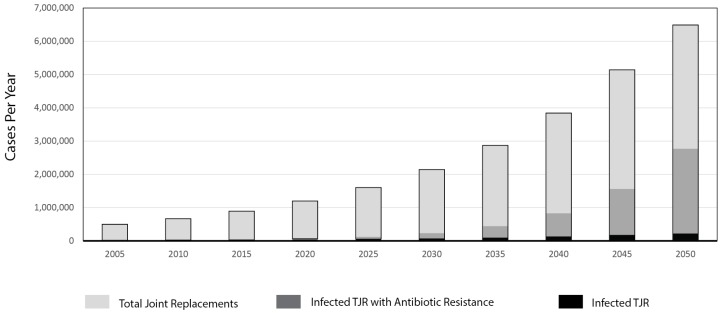
Projected total joint replacements (TJRs) with infection rates with and without the development of antibiotic resistance. Growth rate for TJRs was 6% annual growth and the growth rate for antibiotic resistance was 7% conforming with published numbers [9,16,18].

Periprosthetic Joint Infection (PJI): Despite the clinical success of Total Joint Replacements (TJR) in relieving pain and improving quality of life, up to 10% of implants will fail early (<10 years), requiring a revision procedure to remove the original device and infected bone and replace components [1]. Infection is responsible for 15% of total hip revision surgeries and up to 25% of total knee revisions [18]. A steady increase in TJR procedures (Figure 1) in conjunction with finite infection rates (as low as 1%–2% to as high as 4%–12% [4]), increased lifelong risk of implant bacterial seeding, the rising tide of antibiotic resistant bacteria, and better microbial detection methods have culminated in an increase in the absolute number of patients suffering from acute or chronic osteomyelitis [2,3,19] (Figure 1). Unfortunately, implant removal and hardware replacement drives the risk of infection to a staggering 20%–30% [6,7]. These grim statistics culminate in a significant loss of life (over 1000 deaths per year), many a direct result of the growing frequency of antibiotic resistant bone infections. Moreover, the economic burden for surgically addressing periprosthetic infections (PIs) with revision TJRs is calculated to be 5.3–7.2 times higher than that of primary TJR operations [8] (each revision surgery is estimated to cost $42,000–$56,000 (US) [9]). This amounts to $750 million in insurance and patient costs to treat spine, knee and hip infections and nearly $250 million in hospital losses yearly [7]. Independent biomaterial- or pharmaceutical-based approaches to implant-centered infection are inadequate to combat osteomyelitis. Instead, using an osseo-integrating wound and bone defect filler material as a local delivery vehicle, endowing it with a rate-controlling polymer membrane, may mitigate implant-associated infection and consequent bone destruction. Utilizing principles of intelligent design, an effective, locally deployable, polymer-control antibiotic delivery vehicle can be developed.

Local controlled-release antibiotic delivery to bone: Although osteomyelitis—a serious bone infection accompanied by inflammation, cytokine up-regulation, and bone resorption—is increasingly linked to antibiotic resistant bacteria, current clinical tools to address it are tedious, costly, and often ineffective, compromising the patient’s overall bone health, healing, and recovery. Systemic antibiotic prophylaxis is considered the current clinical standard of care; however, studies are lacking to support this approach [11]. While considered generally effective, problems with systemic antibiotic delivery include systemic side effects and low antibiotic concentrations at the local site of infection, unintentionally favoring antibiotic resistance [10,12]. Bone’s limited vascular supply and the presence of sequestra or void space [14] compounds the problem, inhibiting both delivery of systemic antibiotics and compromising host defenses [4,7]. Sequestra present a favorable, inert environment for harboring bacteria and allowing their unmitigated persistence in the protected avascular wound space [14]. Bacterial persistence in the protected avascular space can lead to progressive acute and chronic osteomyelitis. Alternatively, localized delivery of antibiotics directly to the site of infection is commonly embodied by (1) surgical debridement with an antibiotic solution [7]; (2) application of antibiotic solutions to bone grafts by soaking; and (3) implantation of antibiotic-loaded bone cements. Given that over 500,000 orthopedic procedures annually use bone graft materials to restore lost bone mass and support the tissue bed for implantation [4,5,20], utilizing bone graft void filler (BVF) as an implanted drug delivery vehicle represents a viable strategy to treat PJI.

Controlled drug delivery directly to bone presents an unsolved pharmacokinetic challenge. A strictly biomaterials-based approach has proven inadequate to address infection [17]; improved approaches to implant-centered infection must integrate and exploit local, rate-controlled antimicrobial delivery with appropriate bone defect filler materials. Without a controlled delivery strategy on-board, current antibiotic loaded orthopedic graft materials (antibiotic loaded bone cement (ALBC), OsteoSetT (Wright Medical, Memphis, TN, USA) release their antibiotic payloads too quickly (i.e., 1–4 days), often leaching insufficient antibiotic and serving as a nidus of infection. Additionally, most of these do not support host bone ingrowth [13,21,22]. Previously, we endowed BVF with an antibiotic-releasing polymer barrier capable of releasing its payload in vitro and in vivo for up to eight weeks [23,24,25] as a moldable solid composition. Unfortunately, as with many other antibiotic-releasing BVF devices, our device did not allow host bone ingrowth. Additional efforts proved that host bone ingrowth can be enhanced by intimal contact with the BVF [26]. Thus, designing a space-filling BVF putty with a custom polymer composition to control local antibiotic delivery provides a significant advantage [27,28].

## 2. Materials and Methods

### 2.1. Custom Polymer Synthesis

The synthesis of monomers and polymers is based on previously described procedures [29,30].

**Figure 2:**
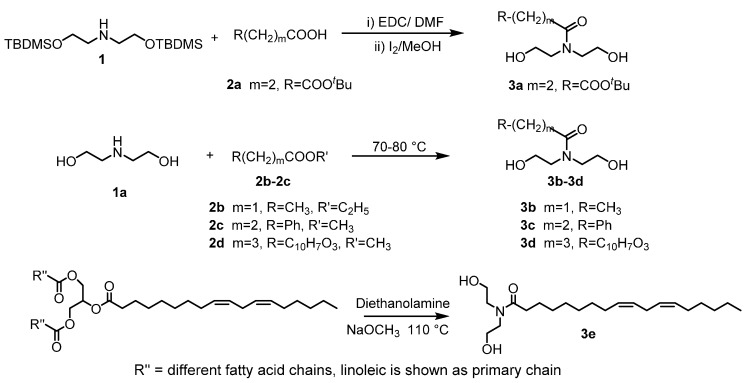
Synthesis of diols with different pendant groups.

Synthesis of 3a (Figure 2): Mono-*tert*-butyl protected succinic acid (5.57 g, 32 mmol), 1-Ethyl-3-(3-dimethylaminopropyl)carbodiimide HCl (EDC, 6.60 g, 34.4 mmol) and anhydrous Dimethyl formamide (DMF, 30 mL) were added into a 250 mL flask and the mixture was stirred for 10 min in an ice bath. To this activated carboxylic acid, compound 1 (8.81 g, 24.6 mmol) dissolved in anhydrous DMF (5 mL) was added and the reaction was stirred overnight. After reacting, DMF was removed under vacuum and the product was extracted with Ethyl acetate (EtOAc, 150 mL). The organic layer was washed with water (50 mL, 1×), saturated NaHCO_3_ solution (50 mL, 1×) and brine (50 mL, 1×) and dried over anhydrous Na_2_SO_4_. The product was purified by column chromatography (15% EtOAc and 85% Hexane).

Yield: 70%: ^1^H NMR (300 MHz, Chloroform-d) ppm 0.04–0.06 (m, 12H) 0.88–0.90 (m, 18H) 1.45 (s, 9H) 2.56 (t, *J* = 6.74 Hz, 2H) 2.67 (t, *J* = 6.58 Hz, 2H) 3.48 (t, *J* = 5.70 Hz, 2H) 3.56 (t, *J* = 5.86 Hz, 2H) 3.74 (t, *J* = 5.70 Hz, 4H).

Deprotection of TBDMS (*t*-butyl dimethyl silane) was performed by reaction with iodine (20% weight of product) in MeOH (15 mL) overnight. Na_2_S_2_O_3_ was added dropwise to quench the reaction until the solution turned from brown to colorless. The solvent was removed under vacuum and the product was extracted with CH_2_Cl_2_ (50 mL, 3×) and dried over anhydrous Na_2_SO_4_. The product was purified via column chromatography (MeOH-CH_2_Cl_2_ gradient solvent system, from pure CH_2_Cl_2_ to 5% MeOH and 95% CH_2_Cl_2_).

Diol 3a: Yield: 61%, ^1^H NMR (300 MHz, Chloroform-d) ppm 0.04–0.06 (m, 12H) 0.88–0.90 (m, 18H) 1.45 (s, 9H) 2.56 (t, *J* = 6.74 Hz, 2H) 2.67 (t, *J* = 6.58 Hz, 2H) 3.48 (t, *J* = 5.70 Hz, 2H) 3.56 (t, *J* = 5.86 Hz, 2H) 3.74 (t, *J* = 5.70 Hz, 4H).

To synthesize diols from ester derivatives of functionalized carboxylic acids (Figure 2), diethanolamine 1a (2 equivalents) and the ester derivative of functionalized carboxylic acid (1 equivalent) were mixed and refluxed in a flask at 75 °C overnight. The product was subsequently purified by column chromatography (MeOH–CH_2_Cl_2_) and completely dried in high vacuum line.

Diol 3b: Yield = 76%, ^1^H NMR (300 MHz, CDCl_3_): ppm 1.16 (dd, 3H, *J1* = 7.61 Hz, *J2* = 7.32 Hz), 2.39–2.50 (m, 2H), 3.50–3.59 (m, 4H), 3.78–3.90 (m, 4H).

Diol 3c: Yield = 63%, ^1^H NMR (300 MHz, CDCl_3_) ppm 2.65–2.74 (dd, *J*1 = 7.61 Hz, *J*2 = 8.20 Hz, 2H), 2.95–3.00 (dd, *J*1 = 7.99 Hz, *J*2 = 8.20 Hz, 2H), 3.42 (t, *J* = 4.98 Hz, 2H), 3.54–3.57 (dd, *J*1 = 4.39 Hz, *J*2 = 4.98 Hz, 2H), 3.70 (t, *J* = 4.98 Hz, 2H), 3.85 (t, *J* = 4.68 Hz, 2H), 7.21–7.32 (m, 5H).

Diol 3d: ^1^H NMR (300 MHz, Chloroform-d) ppm 2.2 (M, 2H) 2.4 (d, *J* = 1.2 Hz, 3H) 2.65 (t, *J* = 7.0 Hz, 2H) 3.53–3.62 (m, 4H) 3.81 (t, *J* = 5.3 Hz, 2H) 3.91 (t, *J* = 5.2 Hz, 2H) 4.12 (t, *J* = 6.0 Hz, 2H), 6.14 (d, *J* = 1.2 Hz, 1H) 6.82–6.89 (m,2H) 7.50 (d, *J* = 8.8 Hz, 1H).

To synthesize the soybean oil monomer, diethanolamine (31.5 g, 0.3 mol) was added into a 500 mL round bottom flask. Subsequently, NaOCH_3_ (0.8 g, 14.8 mmol) was added and stirred at 110 °C until completely dissolved. Soybean oil (43.6 g) was added dropwise via funnel over 30 min. After addition, the reaction was stirred for another one hour at 110 °C under vacuum. EtOAc was added to dilute the reaction mixture, which was then washed with 15% NaCl solution (3×). The product was purified via column chromatography (5% MeOH and 95% CH_2_Cl_2_).

Diol 3e: ^1^H NMR (500 MHz, Chloroform-d) ppm 5.30–5.41 (m, 2.88H) 3.76–3.85 (m, 5.5H) 3.49–3.85 (m, 4H) 2.76–2.81 (m, 1.29H) 2.37–2.40 (t, *J* = 7.70 Hz, 2.09H) 2.00–2.07 (m, 3.38H) 1.63 (br. s., 2.11H) 1.26–1.1.39 (m, 18H) 0.87–0.99 (m, 3H).

**Figure 3:**
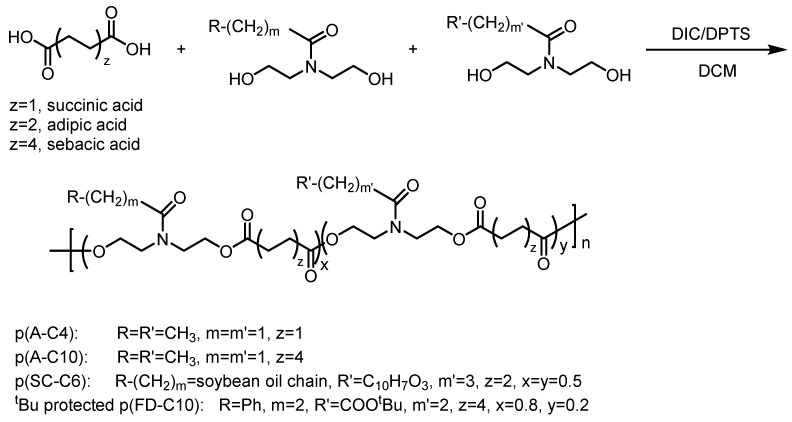
Synthesis of pendant functionalized polyesters.

Polyesterification of functionalized diols and diacids (Figure 3) was accomplished by adding the functionalized diol (1 equivalent), diacid (1 equivalent) and 4-(Dimethylamino)pyridinium 4-toluenesulfonate (DPTS, 0.4 equivalent) into a flask, and the system was evacuated and backfilled with N_2_ (3×). Anhydrous CH_2_Cl_2_ (2 mL for 1 mmol of diacid) was syringed into the reaction flask. The mixture was homogenized by warming to 40 °C for 1–2 min. Subsequently, the mixture was cooled on ice and diisopropylcarbodiimide (DIC) (3 equivalents) was added dropwise by syringe. The mixture was stirred for 48 h at room temperature. The polymer was purified by precipitation from cold *iso*-propanol/methanol or dialysis against MeOH and dried under vacuum. Total equivalents of diols were equivalent to that of diacid if more than one functionalized diol was used in the reaction.

**p(A-C4):** ^1^H NMR (300 MHz, Chloroform-d) ppm 1.15 (t, *J* = 7.46 Hz, 3H) 2.39 (d, *J* = 7.46 Hz, 2H) 2.62 (br, 4H) 3.60–3.70 (m, 4H) 4.22–4.26 (m, 4H). *M*_n_ = 60 KDa, polydispersity index (PDI) = 1.1, *T*_d_: 238 °C, *T*_g_: 5.3 °C.

**p(A-C10):** ^1^H NMR (500 MHz, Chloroform-d) ppm 1.14 (t, *J* = 7.34 Hz, 3 H) 1.29 (br. s., 8 H) 1.56–1.64 (m. 4 H) 2.22–2.35 (m, 4 H) 2.39 (q, *J* = 7.34 Hz, 2 H) 2.66–2.77 (m, 1 H) 3.58–3.64 (m, 4 H) 4.18–4.26 (m, 4 H) *M*_n_: 78 KDa, PDI: 1.19, *T*_d_: 283 °C, *T*_g_: −32 °C.

p(SC-C6): ^1^H NMR (300 MHz, Chloroform-d) ppm 0.87 (d, *J* = 6.8 Hz, 3H) 1.27 (d, *J* = 18.4 Hz, 16H) 1.59 (s, 15H) 2.04 (q, *J* = 6.2 Hz, 3H) 2.16 (t, *J* = 6.0 Hz, 2H) 2.39–2.30 (m, 12H) 2.59 (t, *J* = 7.0 Hz, 2H) 2.76 (t, *J* = 6.0 Hz, 1H) 3.61 (dt, *J* = 11.0, 5.5 Hz, 8H) 4.11–4.08 (m, 2H) 4.19 (d, *J* = 5.0 Hz, 7H) 5.34 (d, *J* = 5.5 Hz, 3H), 6.11 (s, 1H) 6.87–6.80 (m, 2H) 7.49 (d, *J* = 8.6 Hz, 1H) *M*_n_: 11.9KDa, PDI: 2.13, *T*_d_: 320 °C, *T*_g_: −20 °C.

Finally, the tBu-protected p(FD-C10) was deprotected by dissolving the polymer (1 g) in regular CH_2_Cl_2_ (6 mL), trifluoroacetic acid (TFA) (3 mL) and triisopropylsilane (50 μL) and stirred for 2 h. After the stirring incubation, TFA and CH_2_Cl_2_ were removed under vacuum and the polymer was precipitated into diethyl ether twice to obtain pure polymer.

p(FD-C10): ^1^H NMR (500 MHz, Chloroform-d) ppm 1.25–1.36 (m, 8H) 1.53–1.65 (m, 4H) 2.22–2.35 (m, 4H) 2.64–2.76 (m, 2.4H) 2.94–3.01 (m, 2.4H) 3.52 (t, *J* = 5.87 Hz, 2H) 3.58–3.69 (m, 2H) 4.12 (t, *J* = 5.75 Hz, 2H) 4.18–4.26 (m, 2H) 7.16–7.24 (m, 2H) 7.24–7.31 (m, 2H) *M*_n_: 40KDa, PDI: 1.34, *T*_d_: 273 °C, *T*_g_: −16 °C.

### 2.2. Polymer Characterization

Structures of small molecules and polymers were confirmed by 300 and 500 MHz Nuclear magnetic resonance (NMR) proton spectra via a Varian NMRS instrument (Palo Alto, CA, USA). Deuterated chloroform was used as a solvent. Chemical shifts, δ (ppm), were referenced to the residual proton signal.

The molecular weight and polydispersity index for each custom polymer was measured via gel permeation chromatography (GPC). GPC analysis in tetrahydrofuran (THF) was performed on a Waters 150-C Plus instrument (Milford, MA, USA) equipped with refractive index and light scattering detector. Polystyrene was used as a standard. GPC analysis in DMF was performed on an HLC-8320 instrument (King of Prussia, PA, USA) equipped with refractive index and UV detectors using polystyrene as a standard. The flow rate of the eluent was 1 mL/min for both GPC instruments.

The polymer’s decomposition temperature was measured by thermal gravimetric analysis (TGA) with a TA Q500 thermal gravimetric analysis instrument (TA Instruments, New Castle, DE, USA). Each polymer was heated in N_2_ from room temperature to 600 °C with a heating rate of 10 °C /min. Alternatively, the glass transition temperature was tested by differential scanning calorimeter (DSC) using TA Q2000 differential scanning calorimeter (TA Instruments, New Castle, DE, USA) with heating and cooling rates set at 10 °C/min.

### 2.3. Putty Formulation

Antibiotic-loaded bone void filling (ABVF) putties were fabricated similarly to our previous molded formulations [23,24,25] with a couple of critical distinctions to produce a material with a putty-like consistency. Four of the putty formulations contained ProOsteon 500R™ (BVF, Biomet, Irvine, CA, USA) morsalized and sieved to between 150 and 425 micrometers; a combination of polymer binders Poly(d,l-lactide-*co*-glycolide) (PLGA-50:50, Sigma Aldrich, St. Louis, MO, USA) dissolved in *N*-Methyl-2-pyrrolidone (NMP, Fisher, Pittsburg, PA, USA), Poly ethyleneglycol (PEG-5kD, Fluka, St. Louis, MO, USA); and polycaprolactone (PCL-10kD); calcium chloride as a poragen, and vancomycin. Note that vancomycin from two different manufacturers was used in different formulations: (1) Vancomycin HCl Sterile Lyophilized Powder for Injection (Hospira, Inc., Lake Forest, IL, USA); and (2) Vancomycin HCl; Research Products International (RPI), Mount Prospect, IL, USA. PLGA was dissolved in NMP (200 μL) and added to a homogenous mixture of molten PCL and PEG at ~75 °C. BVF, calcium chloride and vancomycin were mixed into the polymer composition and 20 μL of 1× phosphate buffered saline (PBS) was added to produce a putty-like composite. Additional, ABVF putty formulations were created using custom-made polymers (as described above). Briefly, polymers were heated to ~120 °C and ProOsteon 500R™ (Irvine, CA, USA), calcium chloride, and vancomycin (Hospira, Lake Forest, IL, USA) were added. ProOsteon 500R™ (Biomet, Irvine, USA) is a cancellous bone like, osteoconductive matrix consisting of calcium carbonate and hydroxyapatite. The addition of NMP and PBS were not necessary to create a putty-like composite from the custom polymers. With the exception of formulation 2, which was shaped into a single large sphere, all formulations were made as single batches and partitioned into replicates (as indicated in Table 1) using 2 mm diameter × 1.7 mm depth adhesive silicone isolators to form small ABVF disks. One large sphere was also made for formulation 2. Formulations are detailed in Table 1.

### 2.4. Imaging

Both the small cylindrical disk as well as the larger sphere were imaged using scanning electron microscopy imaging. Additionally, the cylindrical disk was imaged using Micro computed tomography (micro CT) at the NDSU (North Dakota State University) Electron Microscopy Center core facility (Fargo, ND, USA).

#### 2.4.1. MicroCT

The sample was hot glued to a glass rod and placed into a GE Phoenix v|tome| xs X-ray computed tomography system with a 180 kV high power nanofocus X-ray tube xs|180 nf, high contrast GE DXR250RT flat panel detector, and molybdenum target (GE Sensing & Inspection Technologies GmbH, Wunstorf, Germany). One thousand projections were acquired at a voltage of 80 kV and a current of 300 µA. Voxel size was 6.4 µm. Acquired images were reconstructed into a volume data set using GE datos|x 3D computer tomography software Version 2.2 (GE Sensing & Inspection Technologies GmbH, Wunstorf, Germany). The reconstructed volume was then viewed and manipulated using VGStudio Max (Volume Graphics Inc., Charlotte, NC, USA).

#### 2.4.2. SEM

Samples for scanning electron microscopy were cut with a razor blade to expose the interior surfaces, attached to cylindrical aluminum mounts with colloidal silver paint (Structure Probe Inc., West Chester, PA, USA) and coated with gold (Cressington Inc., Redding, CA, USA) or with carbon (Cressington Inc.). Images were obtained with a JEOL JSM-6490LV scanning electron microscope or JEOL JSM-7600F field-emission scanning electron microscope (JEOL USA, Inc., Peabody, MA, USA).

### 2.5. In Vitro Release

Three molded ABVF cylindrical disks from both putty formulations 1 and 3 were released after curing 24 h at 4 °C, whereas the three remaining ABVF disks were released after one week at 4 °C. The ABVF putty cylindrical disk released after 24 h weighed an average of 45.6 mg (STD (standard deviation): 4.925). Cylindrical disks were placed in individual 2 mL microcentrifuge tubes containing 2 mL of PBS for six weeks at a constant temperature of 37 °C. PBS was replaced at 24, 48, and 72 h and every week thereafter for a total of six week as previously described [23,31]. At the end of 6 weeks, the remaining ABVF putty samples were dissolved in 1 mL of dichloromethane and 500 µL water, vortexed for 15 s, and centrifuged for 5 min at 15,000 rpm to separate the aqueous layer. The aqueous layer was then collected, to determine presence of unreleased vancomycin. The extracted PBS samples and residual vancomycin in the aqueous layer were then analyzed to determine the amount of vancomycin released and its bioactivity. Alternatively, the large sphere from the entire formulation 2 batch (710 mg) was placed in a 50 mL conical tube and the drug was released into 5 mL of PBS as described for the smaller cylindrical disks.

### 2.6. In Vitro Vancomycin Release Kinetics

The amount of vancomycin released into PBS or the amount of residual vancomycin left in the device after release was determined using a custom-built detector as previously described [32]. Briefly, a 280 nm wavelength light emitting diode (LED) and a photodiode were mounted in a cuvette holder, machined from aluminum such that light from the LED passed through the chamber and into the photodiode aperture. Light that reaches the photodiode was converted into a voltage and read by an analog to digital converter (ADC). A computer, along with supporting circuitry, controlled the entire system. The amount of vancomycin was calculated based on a standard curve. The data were then fitted to different pharmacokinetic models [33].

### 2.7. Kirby Bauer Zone of Inhibition Assay (ZOI)

A standard Kirby Bauer ZOI assay was performed [24,34]. In addition, 100 µL of release solution from each time point was dried on filter paper disk (6.5 mm diameter) before being placed on Trypticase Soy Agar plates (TSA, Fisher Scientific, Pittsburgh, PA, USA) with an average depth of 4.7 mm [31] and spread with a uniform bacterial field of *Staphylococcus aureus* strain 49230 (12 × 10^7^ CFU). Standard vancomycin concentrations (8 mg/mL serially diluted to 0.0625 mg/mL) and PBS blanks were prepared in same fashion as release solution. Plates were inverted and incubated at 37 °C for up to 20 h before diameter of zone of inhibition was recorded via electronic calipers.

### 2.8. Mechanical Characterization

ABVF constructs from formulation 1 with (*n* = 3) and without (*n* = 3) vancomycin and from formulation 4 (*n* = 2) were assessed for their putty-like characteristics using a Bose 3200 load frame (TA Instruments, New Castle, DE, USA) with supplied compression surfaces. A 225N load cell with Wintest software (TA Instruments, New Castle, DE, USA) was used for data collection. Supplied Bose compression surfaces were used in the compressive tests. However, a custom designed and built fixture was used to test the tackiness of the samples in a tensile lap test. This fixture consisted of two 16 gauge 304 stainless blanks, which were placed in grips. The blank held in the bottom grip had a toggle clamp bolted to it, which provided consistent sample thicknesses. The toggle clamp had a foot, which used a roller bearing to provide clamping force but provide minimal drag to the test. Each sample (26–41 mm^3^) was compressed to create a uniform cylinder of 2.45 mm in diameter and 4.8–8.9 mm in height. Samples were tested on the same day they were fabricated.

#### 2.8.1. Compression Testing

Compression tests were run at a rate of 0.1 mm/s. Samples were pressed in the longitudinal direction while the load frame measured strain and load. True stress, which calculates stress based on a changing area, was calculated by solving for the changing contact area in relation to the changing height, assuming constant sample volume. The average and standard deviation was calculated for each formulation.

#### 2.8.2. Tensile Lap Testing

Each material was compressed in the aforementioned lap-testing fixture. Each sample was placed on the upper stainless plate and the lower plate with toggle clamp was clamped compressing the sample to a thickness of 0.77 mm. Each test was conducted at a rate of 0.1 mm/s.

### 2.9. Statistical Analysis

Averages and standard error mean are reported. Any pairwise comparisons presented were done via ANOVA with significance of α < 0.05.

## 3. Results

**Figure 4:**
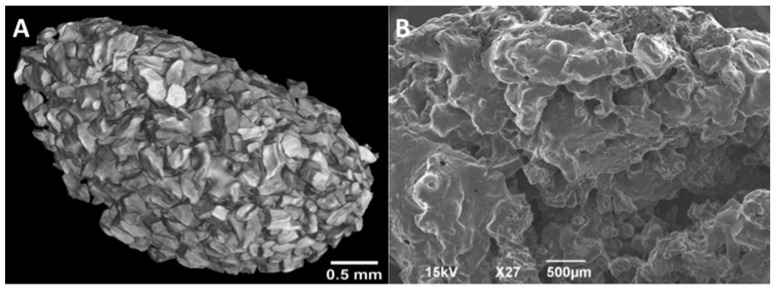
The homogeneity of the composition, percent of Bone Void Filler (BVF), and the porosity of the BVF putty was determined for a model formulation of a Polycaprolactone/poly ethylene glycol/ poly(lactic-*co*-glycolic acid) (PCL/PEG/PLGA) putty using micro computer tomography (CT) on a small cylindrical disk (**A**); and SEM through the center of a large sphere (**B**).

Combinations of common biomedical polymers or four custom-designed, pendant-functionalized polyesters were used to create seven different ABVF putty formulations (Table 1). ABVF putty formulations 1–3 were made as a composite of polymers (i.e., PEG, PCL, PLGA) generally regarded as safe (GRAS) for biomedical applications. To develop an ABVF device with putty-like consistency, previously described methods [23,24,25,26] were modified to include NMP [35]. Alternatively, customized polyesters were synthesized with appropriate pendant groups to enable optimum hydrophobicity or specific interactions to obtain desired release and degradation properties over a 6–8 weeks period. Polymers were synthesized as shown in Figure 2 and Figure 3 and characterized as outlined in Materials and Methods. ABVF putties made with these custom polymers did not require the use of NMP or other co-polymer binders. MicroCT and SEM imaging was done to assess (1) the homogeneity of composition; (2) percentage of mineral (BVF) composition; and (3) qualitative porosity (Figure 4). By adjusting the grey-scale contrast and setting a threshold, the volume of mineral composition was calculated to be ~63.3% from the microCT and SEM images. The actual composition of the formulation was 63%; therefore, the composition seems homogeneous.

**Figure 5:**
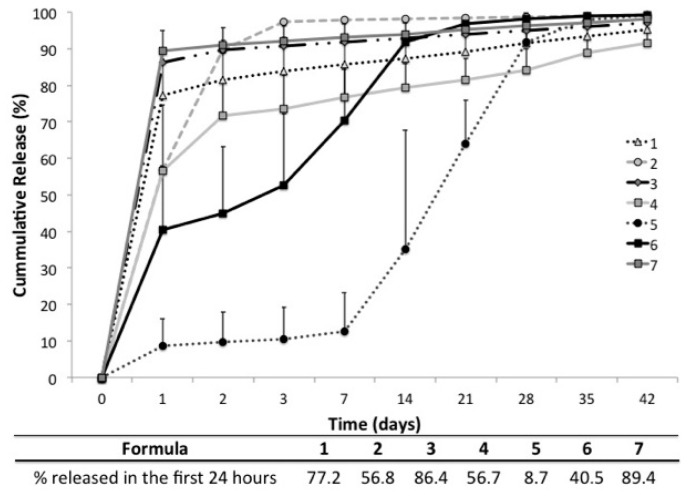
Vancomycin release kinetics for each formulation are shown in the line graph. One of the major distinctions in the formulations is the percent of drug released in the first 24 h. Note that formulation 2 was a larger sphere while all other formulations are smaller cylindrical disks. Standard error mean are shown for each formulation.

In each of the custom polymers (4–6, Table 1), the first two letters indicate the pendant amino acid mimic (A—Alanine, F—Phenylalanine, D—Aspartic acid), whereas the second part denotes the number of carbon atoms in the diacid. Polymer **p(SC-C6**) contains two non-natural pendant groups. The amounts of pendant-functionalized polymers were limiting, and thus all other components of the BVF putty composition were altered to maintain the ratios previously reported [31] in these pilot scale formulation. **p(FD-C10)** had a honey-like consistency and was light sensitive; thus, it was protected from light until it was used in ABVF fabrication. These samples were released or tested the same day and were protected from light during release. Duplicate formulations without vancomycin were made as a negative control (data not shown). There was no bioactivity from any of the control formulations without drugs. Incorporating polymers with different pendant groups and therefore various physical properties as the bulk matrix of an antibiotic-loaded BVF device showed (i) that there was no intrinsic antimicrobial activity for this set of polymers; (ii) **p(FD-C10)**, **p(SC-C6)**, and **p(A-C4)** were all able to release vancomycin for over one month, with **p(SC-C6)** (formulation 5) exhibiting the lowest burst release (Figure 5) and zero order drug release kinetics (Table 2); and (iii) unlike the commercially available polymer formulation, and ABVF formulation with custom polymers displayed evidence of a heterogeneous composition (Table A1).

Vancomycin was released in vitro from a newly developed bone void filling putty that used a varied composition of polymer binders for up to six weeks, a time point considered critical by the orthopedic community for infection prevention [15]. The cumulative percent of vancomycin released was plotted over time (Figure 5). Formulation 1 and formulation 3 only differed in the source of vancomycin incorporated. However, based on the kinetics and bioactivity data, there is no statistical difference between different sources of vancomycin. Formulations 1 and 2 only differ in the geometry and size of the device. The larger spherical device released a little more than half as much drug in the first 24 h as its smaller cylindrical disk counterpart. Additionally, bioactivity of vancomycin released from the larger sphere was extended from three days to two weeks (data not shown), likely as a result of the increased amount of drugs in the larger device. The cumulative percent of vancomycin released at each time point was averaged to produce an average pharmacokinetic curve (Figure 5), which was subsequently evaluated against zero-order, first-order, Korsmeyer–Peppas, Higuchi, and Hixon–Crowell models and the best fit, based on *R^2^* value, was used to determine the mode of release and the importance of geometry and polymer composition (Table 2). A similar analysis was performed for each individual replicate (Table A1). Formulations 1 and 3, which only differ in the source of vancomycin used, showed no significant difference and displayed homogeneous composition as each individual replicate as well as the average all followed the Korsmeyer–Peppas model, which indicated that vancomycin was released from the matrix by both erosion and diffusion. Formulation 2, which only varied in its size and geometry from formulation 1, fit a first-order kinetic model more than any other model. This may indicate that dissolution from the matrix is dependent on the concentration of the dissolving species; however, this supposition remains to be confirmed with additional replicates. The average vancomycin release from formulation 4 is most closely modeled by the Korsmeyer–Peppas equation; however, individual replicates vary between first order and Korsmeyer–Pappas, indicating a lack of homogeneity (Table A1). Formulation 5 closely follows both zero order and Hixon–Crowell with zero order kinetics being slightly favored. This observation again likely reflects that the individual replicates vary between zero order and Hixon–Crowell release models. Nevertheless, this particular formulation provided consistent bioactivity throughout the experimental time course. Formulation 6, which also provided bioactivity throughout the experimental time course, followed both first order and Korsmeyer–Peppas models individually, favoring first order kinetics slightly on average.

**Figure 6:**
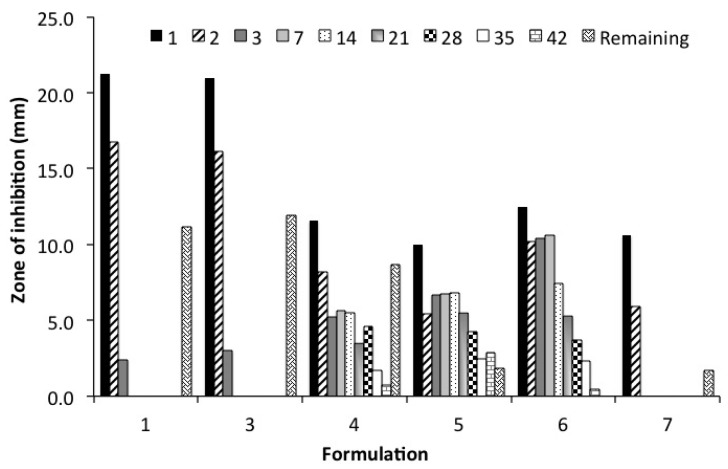
The bioactivity of released vancomycin was determined via a Kirby Bauer zone of inhibition study. Release studies were done for six weeks. At the conclusion of the study, the remaining drug was extracted from the putty. Bioactivity was seen throughout the experimental time course in formulations 4–6.

A standard Kirby Bauer zone of inhibition assay determined the bioactivity of released vancomycin against *S. aureus* over time. Using the “standard” polymer formulation of PCL, PEG, and PLGA that provided sufficient duration of bioactivity from a solid molded BVF form [23,24,25] was insufficient to provide bioactivity past seven days as a BVF putty formulation (Figure 6). However, when the polymer matrix and drug-releasing barrier was a custom, pendant-functionalized polymer, bioactivity could be extended to between 35 and 42 days, dependent on the identity of the polymer and geometry of the putty (Figure 6).

**Figure 7:**
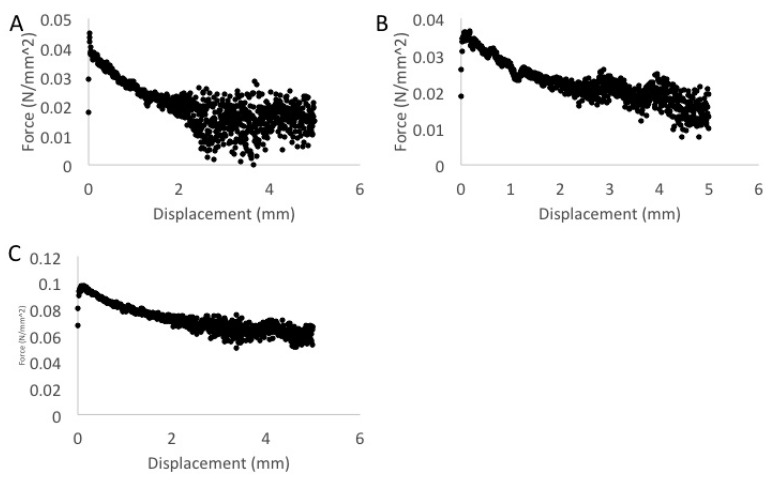
Compression tests of formulation 1 with drugs (**A**) and without drugs (**B**) as well as formulation 4 with drugs (**C**). Notice that the compression behavior of the composition changes quite a bit in the absence of vancomycin and is able to withstand more stress prior to plastic deformation.

**Figure 8:**
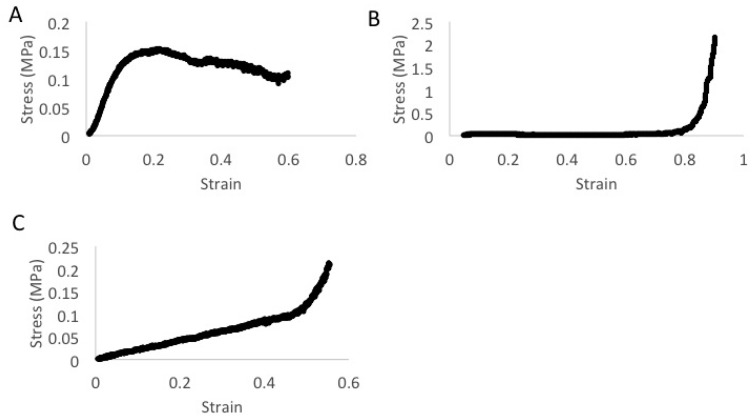
Tensile lap tests of formulation 1 with drugs (**A**) and without drugs (**B**) as well as formulation 4 (**C**) with drug to assess the “stickiness” of the composition. Notice that formulation 4 can withstand almost twice the stress with a corresponding strain.

ABVF from the two most qualitatively disparate formulations based on their stiffness: the commercial polymer formulation (formulation 1) and custom polymer **p(FD-C10)** (formulation 4), were mechanically evaluated as model formulations to characterize the new composition as a putty. Each sample was compressed into a cylinder prior to testing as described in Materials and Methods. A combination of compression (Figure 7) and tensile lap testing (Figure 8) were used to assess the composition’s plastic deformation and adhesion, critical elements in putty-like behavior. Qualitatively, formulation 1 without drugs was very easy to manipulate into shape and was the softest of the three materials, whereas formulation 1 with drugs was more firm and did not deform as easily. Interestingly, despite its viscous honey-like consistency, formulation 4 was the hardest to deform and did not compress into the uniform cylinders as easily. Additionally, formulation 1 with or without the addition of vancomycin kept their shape after being compressed into a cylindrical shape but formulation 4 expanded in a longitudinal direction and did not hold its shape after being removed from the cylindrical mold. Compression of formulation 1 with or without drugs shows how easily deformable the material was, with very low stresses being required to produce deformation. As shown in Figure 7A, formulation 1 with drugs had a proportional, elastic-like, stress-strain relationship to a strain of 0.1. The material then exhibited plastic deformation past strains of 0.1. Surprisingly, compressive behavior changed in the absence of vancomycin and higher strain or 0.8 was required to plastically deform the cylindrical material (Figure 7B). Formulation 4 gave a linear elastic stress-strain relationship to a strain of approximately 0.4 at which time the response changed to plastic deformation (Figure 7C). Tensile lap testing, which assessed the “stickiness” of the composition, showed that formulation 4 was able to maintain almost twice the amount of force when compared to formulation 1 either with or without drugs (Figure 8).

## 4. Discussion

**Figure 9:**
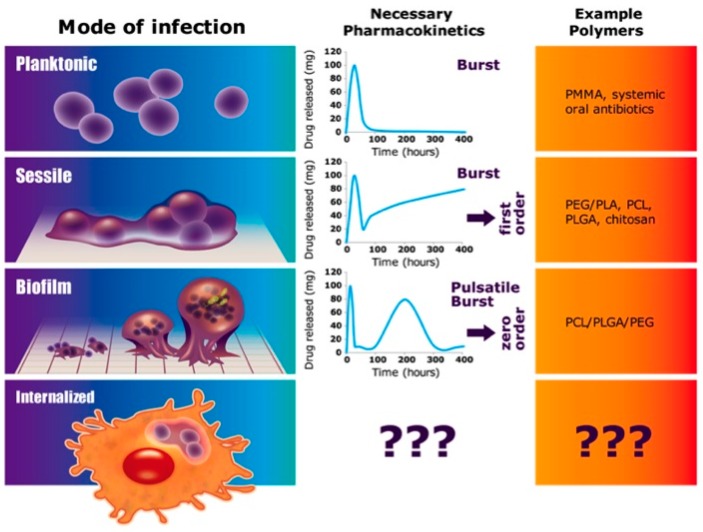
There are four relevant modes of infection related to total joint replacement. Planktonic bacteria-sourced infections can be combatted with simple burst release kinetics such as that provided by bone cements (i.e., Poly(methyl methacrylate)) or systemic antibiotics. After attaching to a surface, sessile bacteria are slightly more difficult to treat and typically require a first-order or zero-order sustained release after the burst. To combat biofilm resident bacteria, a pulsatile burst with zero-order kinetics are thought to be essential. Such sustained release formulations may be provided using combinations of polymers such as that described here. The most challenging orthopedic infections, recurring chronic osteomyelitis, will require distinctly new approaches with new polymer compositions (e.g., pendant-functionalized polymers, etc.).

Osteomyelitis is a complex condition with multiple etiologies that necessitate new, tailorable strategies (Figure 9). Previously, polymer-controlled, antibiotic-releasing, moldable BVF devices proved effective in preventing infection in a rabbit radial defect model. Unfortunately, the solid polymer BVF composite material did not provide intimate contact with the host bone and little cellular infiltration was noted. Thus, a putty composition with similar drug release kinetics was produced. Current bone cements used for orthopedic procedures requiring some mechanical robustness, such as methyl methacrylate in TJR, contain acrylic oligomers or monomers to provide low viscosity pliable composites [36]. The high heat of reaction from these materials can cause tissue necrosis. Alternatively, the use of synthetic polymers such as those previously used to create a solid, moldable, BVF formulation [23,24,25,26] may not be suitable for BVF putties due to their high modulus. Hence, we assessed four custom pendant-functionalized polyesters. Formulations 4 and 5 seemed to have particularly useful pharmacokinetics and will be followed up with a more rigorous statistically significant study. Clinically accepted polymers previously used for BVF drug delivery have a specific set of physical properties that cannot be tuned to the application. Therefore, polymers whose physical and chemical properties can be tailored to the in vivo environment are desperately needed.

Previously, a platform of peptidomimetic polyesters whose physical and chemical properties can be modulated over a wide range of properties was developed [30]. These polyesters are characterized by the presence of “peptide-like” pendant functional groups at every repeat unit, providing a set of tailorable polymers with functionality to suit the application need. The polyesters were designed to mimic the functional diversity of peptides. The currently reported polymers have significantly lower glass transition temperatures (*T*_g_) than the previous polyesters and hence allow fabrication of drug-encapsulated putties at lower temperatures. This is critical for future efforts, which may encapsulate heat sensitive drugs. The polyester, **p(SC-C6)**, with pendant groups having long aliphatic chains lead to polymers with very low *T*_g_. Additionally, the nature of the diacid also influences the *T*_g_ of the polymers. Polymerization of the functionalized diols with long chain diacids such as sebacic acid lowers the *T*_g_ of the polyesters such as **p(A-C10)**. However, unlike polypeptides, the ester backbone ensures hydrolytic degradation and bioresorption, the rate of which can be tuned by the identity of the pendant group. In addition, the polyesters were designed to avoid any hydrogen bonding, which makes the polymers easier to process. The modular synthesis of the polyester platform has enabled the design of such polymers for various applications such as thermoresponsive polymers [29]; polyesters for room temperature 3D printing (accepted, in press); wet adhesives (submitted) and ECM (extracellular matrix) mimetic electron spun matrices for controlled release applications (unpublished).

The physical properties of the polyesters can be engineered by polymerization of appropriately functionalized monomers to provide low-viscosity materials. To achieve the range in properties needed for these studies, the choice of pendant-functionalized diols and the nature of diacids was varied. The identity of the pendant functionalized diol influences the hydrophobic/hydrophilic balance of the polymer and gates the release rate of the encapsulated drug. For example, polyesters having succinic acid (C_4_) will be more hydrophilic and have a higher modulus than the corresponding polyesters made from sebacic acid (C_10_). Therefore, these high molecular weight polymers can be mixed with the bone filler without the use of any organic solvent. The release profiles of a putty-encapsulated therapeutic can be gated utilizing polyester pendant groups (Figure 5 and Figure 6) and provide altered pharmacokinetic and bioactivity profiles, unattainable using single composition common biomaterials such as PCL or PLGA. Importantly, in each formulation 10% *w*/*w* of vancomycin was added. Thus, the amount of vancomycin varied with the weight of the BVF composite device. However, with the exception of the larger formulation 2 sphere, all replicates weighed roughly 50 mg, providing an accurate comparison of the pharmacokinetics. Nevertheless, variation in the amount of vancomycin incorporated cannot be completely ruled out as a contributing factor to the varied pharmacokinetic profiles seen, particularly in light of the apparent heterogeneity of certain formulations.

Using a mix of commercial polymers to create an ABVF putty allowed for a good comparison with previously formulated moldable ABVF fabricated from the same polymer composition. In vitro pharmacokinetics from the moldable ABVF provided antimicrobial activity for up to six weeks [24,26], whereas the analogous composition formulated as a putty only provided effective antimicrobial activity for three days (Figure 6). Although the mechanical criteria to identify a composite as a putty is not clear, putties are generally agreed to have temperature dependent viscoelastic properties and undergo plastic deformation. For the purpose of this study, a putty is defined by its low stress/low strain plastic deformation as well as a “tacky” or “sticky” texture. Due to the high deformability of each sample, true stress was used as opposed to engineering stress as it was determined to be more accurate since the cross sectional area of the ABVF changes under stress. Although stress-strain curves in each of the three samples differed (Figure 7), each material exhibited putty like behavior. Samples required relatively low stress to result in measurable strain. Formulations 1 and 4 had the most disparate properties of all formulations; thus, they were evaluated to (1) confirm that they should be classified as putty and (2) define protocols and procedures for putty characterization. Importantly, in tensile tests of formulation 1 with and without drugs, the force fluctuated a lot in displacements above 2. Additionally, the maximum force at the beginning of the test seemed to drift, necessitating additional replicates. Nevertheless, the methodology was able to reveal differences and indicated that the putty used in formulation 4 was stickier (Figure 8) than the polymer combination used in formulation 1.

The PCL/PEG/PLGA composition seemed susceptible to the effect of geometry, as evidenced by a shift from the Korsmeyer–Peppas model (formulations 1 and 3) [37] to a first-order kinetic model (formulation 2) although even this *R^2^* value is not high and may indicate a mixed mode of release, not uncommon in composite polymers. The Korsmeyer–Peppas model indicates release of the drug from a matrix by a combination of diffusion and erosion. The Higuchi model describes the release of a drug from a matrix tablet by diffusion, whereas the Hixson–Crowell model describes release by erosion. Based on the average of formulation 5, a zero-order release model, which is typically associated with long-duration sustained drug release, is slightly favored, but the Hixson–Crowell model is also closely followed; this closely follows the disparity between individual replicates, which indicate that despite efforts to create a homogenous composition, there was variability. Variability was a common issue for the replicates of formulation 6 as well, which, on average, slightly favored a first-order release model. Additional replicates are necessary to verify which model is more closely followed. Additionally, future efforts must control heterogeneity of composition. Formulations 4 and 7 clearly followed the Korsmeyer–Peppas model more closely.

Using a combination of confirmed and in silico derived models is a powerful approach in controlled-drug delivery formulation development, providing a mechanism to consider each element of the composition (i.e., drug, polymers and additives) to attain a particular pharmacokinetic profile by theoretical means [38]. Unfortunately, even though they are useful to describe drug release kinetics in terms of a classic pharmacokinetic model, these theoretical models lack predictive use. Alternatively, using in silico tools such as Quantitative Structure–Activity Relationship (QSAR) analysis and other molecular modeling tools [39,40,41] (e.g., Schrodinger, Monte Carlo simulations, Molecular Docking, etc.) to quantitatively correlate relationships between trends in chemical structure alterations and respective changes in biological endpoints (i.e., which chemical properties are determinants of biological activities), the number of compounds to be synthesized can be minimized by selecting the most promising candidates [42,43,44]. In silico modeling of drug release kinetics may streamline formulation optimizations to address the most challenging clinical cases of TJR-associated infection (Figure 9) where *S. aureus* enters the osteoblast to avoid antibiotics and the host immune system. Currently, in silico efforts are underway using Schrodinger software (‎New York, NY, USA) to create relevant polymer models of vancomycin release.

## 5. Conclusions

The work presented here represents a logical extension of our previous moldable antibiotic-releasing, polymer controlled BVF device [24]. By including either NMP or using low *T*_g_ custom polymers, a promising putty-like composition was created to locally deliver bioactive vancomycin over 42 days, ultimately facilitating effective treatment for orthopedic *S.aureus* infection. Using the strategies presented here, additional polymers can be developed to tailor the pharmacokinetic profile of vancomycin release and begin to tackle the most challenging chronic and invasive infections.

## Figures and Tables

**Table 1:** Formulations presented in this manuscript. The source of vancomycin is noted in the last column. All formulations had the same ratio of components in the final formulation. The first two letters of the custom polymers (4–6) indicate the amino acid mimic (A—Alanine, F—Phenylalanine, D—Aspartic acid), whereas the second part denotes the number of carbon atoms in the diacid. The polymer p(SC-C6) contains two non-natural pendant groups.

Formulation (number of replicates)	Geometry	Polymer(s)	*N*-Methyl-2-pyrrolidone (NMP)	CaCl_2_	Vancomycin (10% *w*/*v*)
1 (*n* = 6)	Disk	PLGA/PCL/PEG	X	X	RPI
2 (*n* = 1)	Sphere	PLGA/PCL/PEG	X	X	RPI
3 (*n* = 6)	Disk	PLGA/PCL/PEG	X	X	Hospira
4 (*n* = 4)	Disk	**p(FD-C10)**		X	Hospira
5 (*n* = 2)	Disk	**p(SC-C6)**		X	Hospira
6 (*n* = 3)	Disk	**p(A-C4)**		X	Hospira
7 (*n* = 2)	Disk	**p(A-C10)**		X	Hospira

**Table 2:** The amount of vancomycin was plotted against time on the *x*-axis. The average of each formulation was then fitted to each of the common pharmacokinetic models to identify the best fit.

Formulation	Zero-order (*R*^2^)	First-order (*R*^2^)	Korsmeyer-peppas (*R*^2^)	Higuchi (*R*^2^)	Hixon-crowell (*R*^2^)
1	0.15	0.45	0.93	0.30	0.28
2	0.26	0.61	0.51	0.46	0.45
3	0.14	0.42	0.88	0.29	0.25
4	0.28	0.68	0.92	0.45	0.52
5	0.92	0.88	0.86	0.84	0.91
6	0.70	0.97	0.96	0.88	0.90
7	0.16	0.59	0.96	0.32	0.33

## Abbreviations

The following abbreviations are used in this manuscript:

PCL	Polycaprolactone
PLGA	Poly(d,l-lactide-*co*-glycolide)
NMP	*N*-Methyl-2-pyrrolidone
PEG	Polyethyleneglycol
BVF	Bone graft void filler
ABVF	Antibiotic-eluting bone void filler

## Appendix

**Table A1:** The amount of vancomycin was plotted against time on the *x*-axis. Individual replicates of each formulation, indicated by the letter, were then fitted to each of the common pharmacokinetic models to identify the best fit according to *R^2^*.

Formulation	Zero-order (*R*^2^)	First-order (*R*^2^)	Korsmeyer-peppas (*R*^2^)	Higuchi (*R*^2^)	Hixon-crowell (*R*^2^)
1-A	0.14	0.45	0.92	0.29	0.26
1-B	0.34	0.66	0.74	0.54	0.54
1-C	0.14	0.41	0.94	0.29	0.26
1-D	0.16	0.51	0.98	0.32	0.33
1-E	0.14	0.45	0.93	0.29	0.26
1-F	0.14	0.45	0.96	0.29	0.26
2	0.26	0.52	0.51	0.46	0.41
3-A	0.14	0.45	0.90	0.29	0.27
3-B	0.15	0.39	0.93	0.30	0.26
3-C	0.14	0.40	0.97	0.29	0.24
3-D	0.14	0.42	0.96	0.29	0.25
3-E	0.14	0.47	0.63	0.29	0.27
3-F	0.13	0.46	0.94	0.28	0.24
4-A	0.49	0.78	0.76	0.69	0.69
4-B	0.27	0.71	0.83	0.43	0.53
4-C	0.63	0.83	0.71	0.81	0.76
4-D	0.17	0.57	0.82	0.33	0.37
5-A	0.89	0.84	0.77	0.80	0.86
5-B	0.92	0.94	0.93	0.96	0.98
6-A	0.81	0.98	0.95	0.92	0.92
6-B	0.32	0.74	0.84	0.53	0.59
6-C	0.67	0.99	0.96	0.87	0.91
7-A	0.15	0.47	0.63	0.29	0.27
7-B	0.13	0.46	0.94	0.28	0.24
